# The effect of prenatal education on the fear of childbirth: A systematic review and meta-analysis

**DOI:** 10.1007/s00737-025-01635-5

**Published:** 2026-02-10

**Authors:** Zekiye Karaçam, Priscilla Ofei, Gülçin Uzunoğlu, Gizem Güneş Öztürk

**Affiliations:** 1 Faculty of Health Sciences, Division of Midwifery, Aydın Adnan Menderes University, Aydın, 09100 Turkey; 2 Newham University Hospital, London, UK; 3 Manisa Mental Health and Diseases Hospital, Manisa, Turkey

**Keywords:** Education, Prenatal, Childbirth Classes, Birth, Fear, Meta-Analysis

## Abstract

**Purpose:**

To evaluate the effect of prenatal education on the fear of childbirth among pregnant women based on previously conducted studies.

**Methods:**

A systematic review and meta-analysis of randomized controlled trials and quasi-experimental studies was conducted following the PRISMA guidelines. The data were pooled through meta-analysis. ROBINS-I and RoB2 were used to assess the quality of the studies. The GRADE approach was used for evaluating the certainty of evidence.

**Results:**

The meta-analysis included 28 studies and the total sample size of the studies was 3073. The results showed that statistically, prenatal education significantly reduced the fear of childbirth during both the antepartum and postpartum period (SMD: -1.12, z = 9.14, p < 0.001; MD: -24.35, z = 6.18, p < 0.001 respectively). The meta-regression performed indicated that the study design, the course of the COVID-19 pandemic, data collection tools, the countries of the studies and features of education had no effect on the results of fear of childbirth in pregnancy. Moreover, the meta-analyses showed that prenatal education increased the likelihood of vaginal birth and the preference for vaginal birth approximately by two times and three times respectively (OR: 2.00, z = 4.82, p < 0.001; OR: 2.87, z = 3.89, p = 0.001 respectively). The certainty of evidence was low for fear of childbirth during pregnancy, moderate for fear of childbirth in the postpartum period and high for vaginal birth and preference for vaginal birth.

**Conclusion:**

This study revealed that prenatal education was effective for reducing the fear of childbirth and therefore, increasing vaginal births.

**Registration number:**

CCRD42022378547

## Highlights

- Fear of childbirth is a common health problem that negatively affects maternal and new-born health and is particularly associated with increased rates of caesarean section. There is a need for up-to-date information with high evidence level regarding the effectiveness of prenatal education techniques commonly used in the management of fear of childbirth.
- Prenatal education can be used in the management of fear of childbirth.
- This article provides a comprehensive synthesis of current studies, highlighting important information that prenatal education can reduce birth fear in the prenatal and postnatal periods and increase vaginal birth and vaginal birth preference.

## Introduction

Childbirth experience is generally characterised by positive expectations, however, for some women, childbirth or only the idea of it is accompanied by fear. Fear of childbirth (FOC) could affect the normal functioning of women in diverse ways, aggravate to clinical relevance, or even affect crucial decisions related to childbirth (Elsharkawy et al. 2024; Moro et al. 2023; Sanjari et al. 2021). Therefore, it is an important health problem that needs to be carefully evaluated and managed.

The most recent meta-analysis reported a global prevalence of 16% for severe FOC among low-risk women (Sanjari et al. 2021). In recent studies, the prevalence of FOC in pregnant women is reported as 29.2% in Iran (Barat et al. 2023), 11.3% in Egypt (Elsharkawy et al. 2024), 12% in Brazil (Moro et al. 2023), 33.8% and 42.4% in Turkey (Gökçe İsbir et al. 2024; Yılmaz and Can Gür 2021). A study conducted in China reported that 20.8% of women experienced severe FOC before birth and 18.2% after birth (Xu et al. 2024). Additionally, FOC has been reported to be more prevalent with low education status, unemployment and low financial status, younger age, divorce, singlehood, nulliparity, nulliparity, being in the first trimester, planning status pregnancy, previous negative birth experiences, stillbirth, insufficient social support, histories of infertility and psychiatric disorders (Barat et al. 2023; Elsharkawy et al. 2024; Gökçe İsbir et al. 2024; Moro et al. 2023; Sanjari et al. 2021; Yılmaz and Can Gür 2021).

FOC is an emotional stressor that affects the mental health and the wellbeing of pregnant women throughout pregnancy. Being a mental condition and considering its steadily increasing rate, FOC is thought to be a consequential health issue for both Turkey and the world (Sanjari et al. 2021). In a study conducted among pregnant women in a maternity hospital, 45.4% of the women had FOC; the reasons for FOC were the inability of the pregnant woman to trust herself during childbirth and the fear of suffering, caesarean section or episiotomy (Elsharkawy et al. 2024; Johnson et al. 2019). The most significant effect of FOC, perhaps, is its contribution to the ever-increasing caesarean birth rates beyond the World Health Organization’s recommended 15% rate (World Health Organization 2015). Various studies have reported a strong association between FOC and caesarean birth, especially, caesarean section on maternal request (Ensari Altun et al. 2022; Størksen et al. 2015). FOC, as reported by a study, increased the risk of caesarean birth by 5.2 times and prolonged the active phase of labour approximately by 40 min (Sezen and Ünsalver 2018).

Antenatal education is among the interventions reported to be effective in the management of FOC (O’Connell et al. 2021; Webb et al. 2021). Pregnant women need prenatal education in order to cope with the birth process and develop skills related to neonatal care, puerperium and parenting after birth (Yazıcıoğlu and Yavuz 2022). Reasons for FOC among women include presence of an infirmity in the baby, being alone in a foreign environment, concerns about taking a wrong decision related to her own or the baby's health or harming the baby, thoughts of experienced or narrated severe pain, distrust in the health institution and its team, loss of control during the birth process and fear of death (Yazıcıoğlu and Yavuz 2022). In the alleviation of FOC based on reasons such as outlined above, prenatal education is beneficial. Furthermore, to reduce unnecessary caesarean sections, WHO recommends psychoeducation, including information on fear and anxiety, FOC, normalization of individual reactions, stages of birth, hospital routines, delivery process and pain relief, for women with fear of pain (World Health Organization 2018). Management of FOC aims at helping the woman to accept the uncertainties of childbirth, have positive feelings about childbirth, control the fear during pregnancy and help reduce anxiety related to birth (Çiftçi et al. 2022).

Birth preparation education may be carried out individually or in classes; its practice has been well established in developed countries and is being incorporated into routine care at a rapid rate in developing countries today (Buran et al. 2020). Antenatal education models such as Bradley, Dick-Read, Hypnobirthing and Lamaze method are popular in our days and extremely effective, and models such as Birthing From Within, Awareness Based Birth Preparation, Odent Method, Kitzinger Method and Leboyer Method are in use as well (Coşar and Demirci 2012; Walker et al. 2009). The present study focuses on prenatal education in general without emphasis on any education method in particular.

Considering the negative effects of fear of childbirth on mother-baby and family health, especially the increase in caesarean section rates, it needs to be diagnosed and managed very well. The oldest and most widely used approach to the management of fear of childbirth is prenatal education classes. In recent years, it has been observed that these trainings have been enriched with some special techniques such as psychodrama (Bayrı Bingöl et al. 2022), hypnobirthing (Buran and Aksu 2022), solution-focused psychoeducation (Kaya and Guler 2022) and mindfulness (Veringa-Skiba et al. 2022). However, more high-level evidence-based information is needed to demonstrate the effectiveness of these methods. There are some meta-analysis studies in the literature showing the effectiveness of prenatal education approaches in reducing fear of childbirth (Alizadeh-Dibazari et al. 2023; Demirci et al. 2023; Aguilera‐Martín et al. 2021; Yıldız Karaahmet et al. 2023). These studies report that prenatal education reduces fear of childbirth (Alizadeh-Dibazari et al. 2023), improves women's ability to cope with childbirth and have a positive birth experience ( Demirci et al. 2023), the use of additional approaches such as social support and psychoeducation and counselling during the prenatal education process can increase the effectiveness of the education (Aguilera‐Martín et al. 2021), and psychoeducation reduces fear of childbirth, anxiety, and cesarean section rates and increases self-efficacy (Yıldız Karaahmet et al. 2023). With this study, we aimed to reveal the effect of prenatal education on fear of childbirth and vaginal birth and vaginal birth preference during pregnancy and postpartum periods using meta-analysis and the GRADE approach, based on more up-to-date and numerous research results. In addition, this study aimed to reveal information about the design of studies on fear of childbirth during pregnancy and effect size, the COVID-19 pandemic, data collection tools, countries where the studies were conducted, and the effect of the use of additional approaches in prenatal education through meta regression. It is expected that the information obtained will contribute to the development of health service provision related to prenatal education and to the improvement of the birth experiences of pregnant women and maternal-infant health.

### Aim and research questions

This systematic review was aimed at evaluating the effect of prenatal education on the FOC among pregnant women based on previously conducted studies. Research question: (1) What is the effect of prenatal education on the fear of childbirth in the prenatal and postnatal periods? (2) Does prenatal education have an effect on vaginal birth and vaginal birth preference? (3) Does the study design, the COVID-19 pandemic, data collection tools, the country of the studies, and the use of additional approaches in education have an effect on the effect size of prenatal education on fear of childbirth in pregnancy?

## Methods

### Protocol and registration

This systematic review and meta-analysis was conducted following the criteria provided by PRISMA (Preferred Reporting Items for Systematic Reviews and Meta-Analyses) (Page et al. 2021). The protocol of this systematic review was registered to PROSPERO (ID: CRD42022378547).

### Eligibility criteria

Studies were included in this study according to the following criteria (PICOS); Population (P): Pregnant women. Intervention (I): Prenatal education. Comparison (C): Pregnant women who did not receive prenatal education. Outcomes (O): Primary outcomes; fear of childbirth in the prenatal and postnatal periods (fear of birth scale score). Secondary outcomes; type of delivery and option of delivery. Study design (S): Randomized controlled and quasi-experimental studies reporting the effect of prenatal education on the FOC were included in the study. In order to obtain current and up-to-date results, studies published in Turkish and English in the 2017–2024 were included. Studies for which the full text was not available, and the FOC was not measured with a scale as well as qualitative studies and systematic reviews were excluded from this study.

### Information sources and search strategy

Data for the current study were collected in October—December 2022 and updated in September 2024. The literature search for the study was performed on PubMed (including MEDLINE), PsycINFO (All via Ovid SP), EBSCOhost, Web of Science, Scopus, Turkish Medline, Turkish Clinics, and ULAKBIM databases. The keywords used in the scan of the literature were “(prenatal education OR antenatal education) AND fear AND (childbirth OR birth)”. For additional scanning, the reference lists of the studies included in this study and previous reviews on the subject were scanned and additional searches were conducted on Google Scholar.

### Selection process of studies

The studies for this systematic review were selected by the first and second authors independently. Firstly, the results obtained from the scan were examined based on the title and abstract, and the full texts of the studies that were likely to be included in this systematic review were downloaded and collected in a file. Then, the articles to be included in the study were determined after the full texts of these downloaded articles had been examined in accordance with the inclusion criteria. Additional scanning was done during the selection process. Following, the studies obtained by both authors were compared and converted into a single file. In case of differences in opinion among researchers regarding any study, a consensus was reached by discussion.

### Data extraction process

A data extraction tool developed by the Joanna Briggs Institute (JBI) and obtained from the website, with modifications appropriate for the present study, was used to acquire research data. With this data extraction tool, data on the place and year, method, data source, sample size and main findings (FOC, as defined in the studies) of the studies included in the systematic review and meta-analysis was obtained.

### Study risk of bias assessment

The articles included in this systematic review were examined for their methodological quality, using the Cochrane risk-of-bias tool for randomized trials (RoB 2) (Sterne et al. 2019) and ROBINS-I for quasi-experimental studies (Sterne et al. 2016). The quality assessment process was carried out independently by the first and second authors. Then, all three researchers discussed, resolved disagreements, and created a one-sheet report of findings at a meeting.

### Data synthesis methods

Meta-analysis was used for the synthesis of the data in this systematic review. RevMan 5.4.1 (The Nordic Cochrane Centre, Copenhagen, Denmark) was used for the meta-analysis. Heterogeneity among the studies reviewed was assessed using the Cochran Q, χ2 and Higgins I^2^ tests, and an I^2^ > 50% was to indicate significant heterogeneity. I^2^ = 0–40% indicated insignificant heterogeneity, I^2^ = 30–60% indicated moderate heterogeneity, I^2^ = 50%–90% represented substantial heterogeneity and I^2^ = 75–100% indicated considerable heterogeneity (Deeks et al. 2021). Therefore, in the meta-analysis for vaginal birth, a fixed-effects model was used because the heterogeneity was low (I^2^ ≤ 50%), whereas a random-effects model was applied for vaginal birth preference, fear of childbirth during pregnancy, and postpartum outcomes, which showed high heterogeneity (I^2^ > 50%). The random-effects model was applied using the DerSimonian–Laird method. The study data were analyzed using 95% confidence intervals (CIs), with odds ratios (ORs) calculated for categorical variables (vaginal birth and vaginal birth preference), mean differences (MDs) for continuous variables measured with the same instrument (the fear of childbirth in the postpartum period), and standardized mean differences (SMDs) for continuous variables measured with different instruments (the fear of childbirth in pregnancy).

To explain the heterogeneity among studies, a meta-regression analysis was conducted to examine the effects of study design, the course of the COVID-19 pandemic, the countries in which the studies were conducted, data collection tools, and additional educational approaches on fear of childbirth during pregnancy. Since no significant or influential variables were identified in the meta-regression, no subgroup analyses were planned. Meta-regression and publication bias analysis were conducted using the IBM SPSS Statistics. Publication bias was evaluated with a funnel plot and the Egger's regression-based test. All tests were calculated as two-tailed and a p value of < 0.05 was accepted as statistically significant.

### Determining certainty of evidence

The GRADE approach recommended by the Cochrane working group was used for evaluating the certainty of evidence for the critical outcomes of the study (Ryan & Hill 2016; Schünemann et al. 2013). For each outcome, a GRADE evidence profile was drawn up and evidence tables were created. In the creation of the GRADE profile tables, the research design and quality evaluation results of studies reported were taken into account.

## Results

### Study selection results

**Fig. 1:**
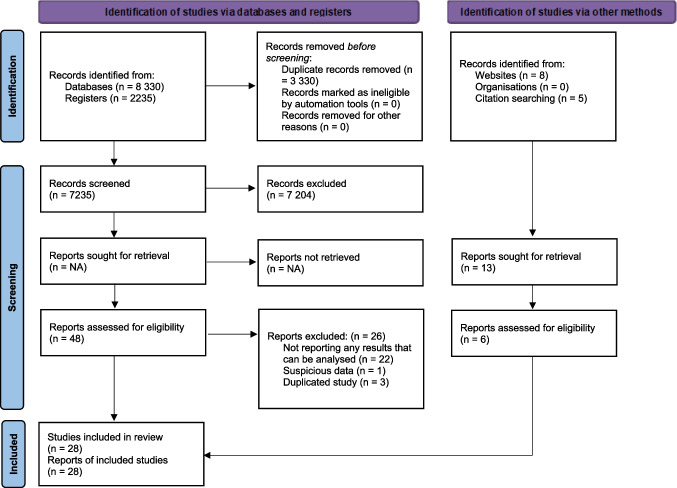
Flowchart of the Study

10 566 records were reached through scanning. After removing duplicates and assessing the obtained records based on title and abstract, 48 articles the full text of which would be examined were identified. The examination of these full text articles yielded 21 articles related with the effect of prenatal education on the FOC which would be included in this systematic review and meta-analysis. Four studies from other sources and three studies from updated searches were added and a total of 28 studies were included in the meta-analysis. The number of studies screened in the systematic review, those eligible and included in the review, along with the studies excluded and reasons for exclusion is given in a PRISMA flowchart (Fig. 1).

### Characteristics of studies and participants

A total of 13 randomised controlled trials and 15 quasi-experimental studies published between 2018 and 2024 were included the present study. While 22 studies were conducted between 2014 and 2021, three studies did not report the year when they were carried out (Buran and Aksu 2022; Hands et al. 2020; Hassanzadeh et al. 2020). The study setting included Turkey (Akın et al. 2018; Aslantekin Özçoban et al. 2022; Bayrı Bingöl et al. 2022; Buran and Aksu 2022; Calpbinici and Özçirpan 2022; Çankaya and Şimşek 2021; Kaya and Guler 2022; Sak et al. 2022; Şimşek 2021; Uludağ et al. 2022; Unutkan 2018; Yazıcıoğlu and Yavuz 2022; Yeşildağ Çelik 2022; Yörük and Acikgoz 2023), Iran (Firouzan et al. 2020; Hassanzadeh et al. 2020; Khojasteh et al. 2022; Moshki et al. 2024; Mousavi et al. 2022; Shaliha et al. 2024), the Netherlands (Veringa-Skiba et al. 2022), the United States of America (Hands et al. 2020), Malawi (Munkhondya et al. 2020), Northern Cyprus (Sarpkaya Güder et al. 2018), Taiwan (Kuo et al. 2022), China (Dai et al. 2021) and Iceland (Swift et al. 2021). The studies were generally carried out in healthcare facilities including hospitals, out-patient clinics, and family health centres. Four studies were conducted with pretest- posttest evaluation without a control group (Akın et al. 2018; Hands et al. 2020; Sak et al. 2022; Yazıcıoğlu and Yavuz 2022). The sample size amounted to 3073 women in total (Experimental: 1258, control: 1310, pre-test and post-test: 505). The age and average age of the participants were generally between 18–36 years (Table 1).

**Table 1:** Characteristics, main findings, and related factors of cross-sectional studies on suicide attempts included in the systematic review

Author (year)/Country	Study Design	Year of Data Collection	Study Location	Sample Size	Mean Age of Patient (years)	Type of Education	Data Collection Tools	Main Outcomes
Akın and Yeşil (2018)/Turkey	Quasi-experimental	2017	Hospital	121 (no control group)	23.73 ± 3.33	Training to reduce fear of birth	WDEQ-A	WDEQ-A scores
Alanya Tosun et al., (2021)/ Turkey	Quasi-experimental	2018- 2020		I: 80 C: 79	I: 26.70 ± 3.71 (20–39) C: 26.61 ± 5.04 (19–41)	Antenatal education	W-DEQ- A	WDEQ-A scores Vaginal birth
Bayrı Bingöl et al. (2022)/Turkey	RCT	2018—2020	Teaching hospital	WDEQ-A I: 61 C: 59 WDEQ-B I: 50 C: 49	I: 26.39 ± 4.05 C: 26.03 ± 3.24	Psychodrama classes	WDEQ-A WDEQ-B	WDEQ-A scores WDEQ-B scores Vaginal birth
Buran and Aksu (2022)/Turkey	RCT	-	Maternity hospital	I: 31 C: 24	I: 29 (20–36) C: 28 (23–34)	Hypnobirthing training	WDEQ-B	WDEQ-B scores Vaginal birth
Calpbinici and Özçirpan (2022)/Turkey	RCT	2019–2020	Out-patient clinic	I: 37 C: 36	18–35	Training programme through the motivational interview method	WDEQ-A WDEQ-B	W-DEQ A scores WDEQ-B scores
Çankaya and Şimşek (2020)/Turkey	RCT	2019	Maternity hospital	WDEQ-A I: 57 C: 59 WDEQ-B I: 55 C: 57	25.8 I: 26.4 ± 3.1 C: 25.3 ± 3.7	Antenatal education	WDEQ-A WDEQ-B	WDEQ-A scores WDEQ-B scores Vaginal birth Preferred vaginal birth
Dai et al. (2020)/China	RCT	2018–2019	Teaching hospital	I: 26 C:30	I: 28.42 ± 2.53 C: 28.20 ± 2.19	Simulation-based childbirth education	WDEQ-A	WDEQ-A scores Vaginal birth Preferred vaginal birth
Firouzan et al. (2020)/Iran	RCT	2019	Antenatal clinics	I: 35 C: 33	İ: 26.27 ± 4.48 C: 25.87 ± 4.58	Psychoeducation	WDEQ-A	WDEQ-A scores Preferred vaginal birth
Hands et al. (2020)/US	Quasi-experimental	–	Community Hospital	207 (no control)	under 36 years	Childbirth classes	Fear and Birth Preference Questionnaire	Fear and Birth Preference Questionnaire scores Preferred vaginal birth
Hassanzadeh et al. (2020)/Iran	Quasi-experimental	2020	20 healthcare complexes	I: 68 C: 68	I: 25.7 ± 4.7 C: 25.8 ± 5.7	Childbirth preparation classes	WDEQ-A	WDEQ-A scores
Kaya and Guler (2022)/Turkey	RCT	2020	Family health centre	SFP: 39 CPT: 40 C: 40	SFP: 27.67 ± 3.71 CPT: 27.80 ± 2.71 C: 27.18 ± 3.99	Solution‐focused psychoeducation	Childbirth Attitude Scale	Childbirth Attitude Scale scores
Khojasteh et al. (2022)/Iran	Quasi‑experimental	2020	Health centre	I: 50 C: 50	I: 16.52 ± 1.43 C: 16.94 ± 1.44	Cognitive-behavioural training	WDEQ-A	WDEQ-A scores
Kuo et al. (2022)/Taiwan	RCT	2018–2019	Teaching hospital	I: 53 C: 53	33.9 ± 4.4	Integrated childbirth education program	WDEQ-A W-DEQ-B	WDEQ-A scores W-DEQ-B scores
Moshki et al. 2024/Iran	Quasi-experimental	2018–2019	Health centre	I: 36 C: 36	30.56 ± 5.3	Face-to-face antenatal education	WDEQ-A	WDEQ-A scores
Mousavi et al. (2022)/Iran	Quasi-experimental	2018	Prenatal Clinic	Social media education (A): 53 In person education (B): 52 C: 50	A: 25.67 ± 4.79 B: 27.59 ± 3.61 C: 26.52 ± 4.35	Childbirth preparatory course	WDEQ-A	WDEQ-A scores Preferred vaginal birth Vaginal birth
Munkhondya et al. (2020)/ Malawi	Quasi- experimental	2018	Community Hospital	I: 35 C: 35	I: 19.83 ± 2.90 C: 20.11 ± 2.70	Companion- integrated childbirth preparation	Childbirth attitude questionnaire	Childbirth attitude questionnaire scores
Özçoban et al. (2021)/Turkey	RCT	2018–2019	Family Health Centre	HL-AE: 53 AE: 56 C: 73	HL-AE: 26.23 AE: 25.86 C: 25.16	Antenatal education for improving health literacy Antenatal education	Fear of Childbirth and The Postpartum Period Scale	Fear of Childbirth and The Postpartum Period Scale score
Sak et al. (2020)/Turkey	Quasi-experimental	2017–2018	Hospital	53 (no control group)	26.9 ± 4.3	Pregnancy Information Classes	Fear of Birth Scale	Fear of Birth Scale scores Preference for vaginal birth
Sarpkaya Güder et al. (2018)/Northern Cyprus	Quasi-experimental	2015–2016	Antenatal clinic	I: 54 C: 54	I: 28.05 ± 2.91 C: 28.00 ± 3.69	Pilates-assisted childbirth preparation training	WDEQ-A	WDEQ-A scores Vaginal birth
Shaliha et al. (2024)/Iran	Quasi‑experimental	2021	Health care centres	I: 34 C: 38	I: 29.24 ± 5.27 C: 27.61 ± 4.40	Antenatal education with scenarios and a booklet	W-DEQ A W-DEQ B	WDEQ-A scores W-DEQ B scores
Şimşek H (2021)/Turkey	Quasi-experimental	2018–2019	Hospital	I: 47 C: 47	I: 27,9 ± 3,6 C: 25,1 ± 4,5	Antenatal education	W-DEQ A W-DEQ B	WDEQ-A scores W-DEQ B scores
Swift et al. (2021)/Iceland	Quasi-experimental	2017–2018	Healthcare clinics	I: 32 C: 60	EAC: 28.3 ± 5.1 AC: 27.9 ± 4.4	Enhanced Antenatal Care	WDEQ-A	WDEQ-A scores
Uludağ et al. (2022)/Turkey	RCT	2021	University hospital	I: 23 C: 21	I: 26.69 ± 4.93 C: 25.66 ± 4.58	Online antenatal education	The Fear of Birth Scale	The Fear of Birth Scale scores
Unutkan (2018)/Turkey	RCT	2016–2017	Antenatal education classes	Antenatal I: 45 C: 44 Postnatal I: 21 C: 25	I: 18–34 C: 18–34	Antenatal education	WDEQ-A WDEQ-B	WDEQ-A scores WDEQ-B Scores Preferred vaginal birth Vaginal birth
Veringa-Skiba et al. (2021)/Netherlands	RCT	2014–2017	Midwifery settings	I: 57 C: 56	I: 33.11 ± 3.92 C: 32.72 ± 3.86	Mindfulness-based childbirth and parenting	W-DEQ-A	WDEQ-A scores
Yazıcıoğlu and Yavuz (2022)/ Turkey	Quasi-experimental	2019	Antenatal clinic	124	27.13 ± 5.07	Antenatal education	W-DEQ-A	WDEQ-A scores
Yeşildağ Çelik (2022)/Turkey	RCT	2021	Family health centre	I: 37 C:36	20–35	Birth preparation training supported by motivational interviewing	W-DEQ A W-DEQ B	WDEQ-A scores W-DEQ B scores Preferred vaginal birth Vaginal birth
Yörük and Acikgoz (2023)/Turkey	Quasi-experimental	2020	Public hospitals	I: 67 C: 66	I: 27.5 ± 4.63 C: 27.89 ± 3.95	Antenatal education	W-DEQ-A	WDEQ-A scores

RCT: Randomised control trial; I: Intervention group; C: Control group; WDEQ-A: Wijma Delivery Expectancy/Experience Questionnaire Version A; WDEQ-B: Wijma Delivery Expectancy/Experience Questionnaire Version B; HL-AE: Antenatal education for improving health literacy; AE: Antenatal education; SFP: Solution‐focused Psychoeducation; CPT: Childbirth preparation training

The Wijma Delivery Expectancy/Experience Questionnaire (WDEQ) was the tool used for measuring FOC in most of the studies; the version A (WDEQ-A) is used to measure FOC during pregnancy in most of the studies whereas the version B (WDEQ-B) is used to measure Fear of Birth Scale (FOC) among women who have experienced childbirth. Nonetheless, to measure FOC during pregnancy, few studies used other tools such as the Fear and Birth Preference Questionnaire (FBPQ) (Hands et al. 2020), the Childbirth Attitude Scale (CAS) (Kaya and Guler 2022) and the Fear of Childbirth and the Postpartum Period Scale (Aslantekin Özçoban et al. 2022).

### Characteristics of the interventions

The interventions offered varied from classic prenatal education to various special education and training (psychodrama classes, hypnobirthing training, simulation-based childbirth education, psychoeducation, solution‐focused psychoeducation, cognitive-behavioural training, companion-integrated childbirth preparation training, prenatal education for improving health literacy, pilates-assisted childbirth, online antenatal education, and mindfulness-based childbirth and parenting, antenatal education with scenarios and a booklet). Three studies had two interventions groups (Aslantekin Özçoban et al. 2022; Kaya and Guler 2022; Mousavi et al. 2022) (Table 1).

### Results for quality assessment

**Fig. 2:**
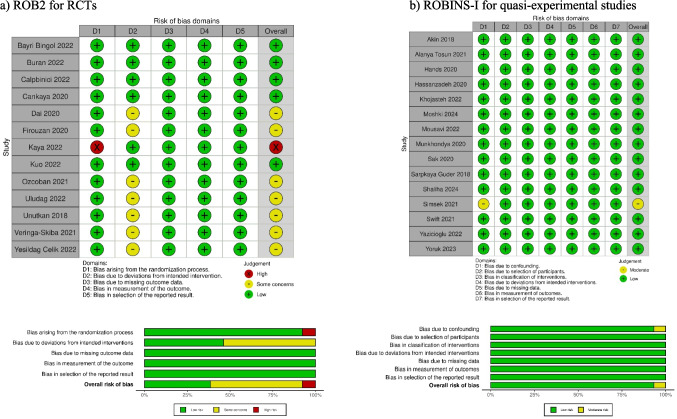
Risk of bias in the studies

Five of the randomized controlled trials had low risk of bias, seven had some concerns, and one had high risk of bias (Fig. 2a). Of the quasi-experimental trials, 14 had low risk of bias and one had moderate risk of bias (Fig. 2b).

## Results for meta-analysis

### The effect of prenatal education on the fear of childbirth

**Fig. 3:**
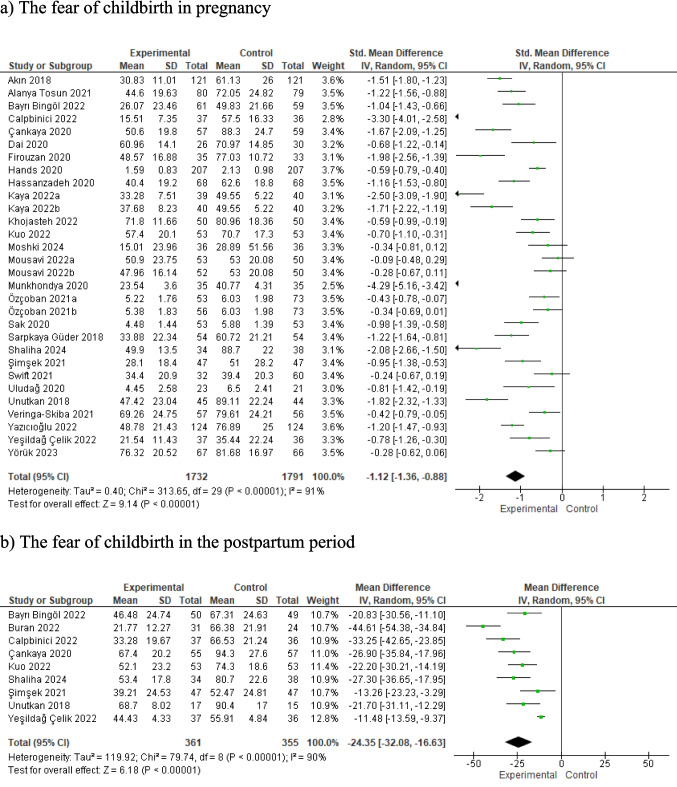
Meta-analysis results for the effect of prenatal education on the fear of childbirth (Kaya 2022, Mousavi 2022 and Özçoban 2021 had two intervention groups and these groups were registered as a and b in the figures)

**Fig. 4:**
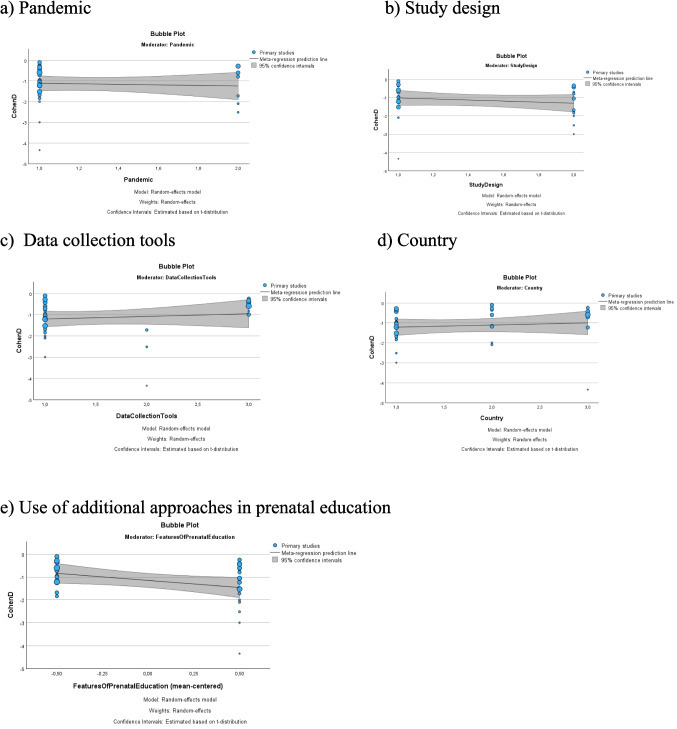
Meta-regression results for fear of birth in pregnancy (a: During pandemic. b: Study design. c: Data collection tools. d: Country of studies for Turkey, Iran, and others. e: Use of additional approaches in prenatal education)

In the present study, in which the results of 27 studies reporting the effect of prenatal education on the fear of childbirth are combined, prenatal education was found to reduce the fear of childbirth statistically significantly (SMD: −1.12, 95%CI: −1.36- −0.88, z = 9.14, p < 0.001, I^2^ = 91) (Fig. 3a). Additionally, The meta-regression performed indicated that the study design, the course of the COVID-19 pandemic, measurement tools used in data collection, the countries where the studies were conducted, and use of additional approaches in prenatal education had no effect on the results of fear of childbirth in pregnancy (t = 0.90, p = 0.377; t = 0.19, p = 851; t = 0.81, p = 0.428; t = 0.23, p = 0.822, t = −1.95, p = 0.062 respectively) (Fig. 4).

Nine studies reported findings on the effect of prenatal education on postpartum fear of childbirth. The meta-analysis based on the results of these studies indicated that prenatal education was effective in reducing the fear of birth in the postpartum period (MD: −24.35, 95%CI: −32.08- −16.63, z = 6.18, p < 0.001, I^2^ = 90) (Fig. 3b). In all the studies examining the fear of childbirth in the postpartum period, the measurement tool used was WDEQ-B. Meta-regression was not performed because only two studies were quasi-experimental (Shaliha et al. 2024; Şimşek 2021), one study was conducted from different country (Kuo et al. 2022) and one study was conducted during the pandemic period (Yeşildağ Çelik 2022).

#### The effect of prenatal education on the mode of birth and the preferred mode of birth

**Fig. 5:**
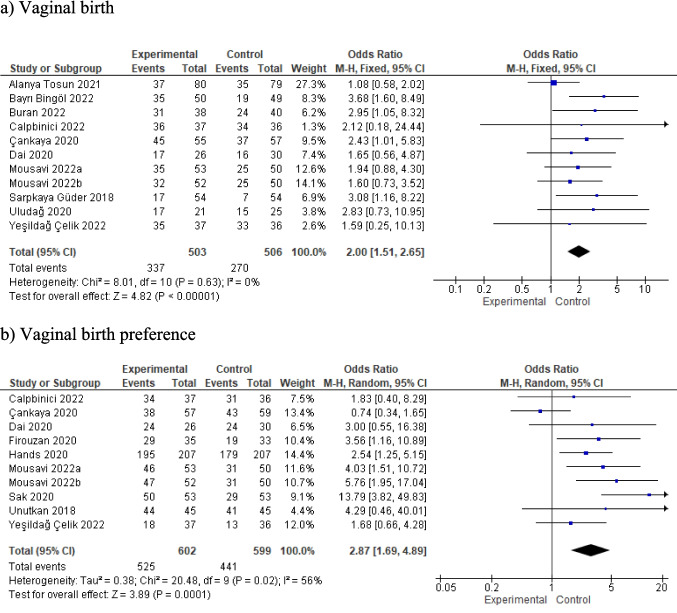
Meta-analysis results for the effect of prenatal education on vaginal birth and vaginal birth preference

Ten studies included in this meta-analysis reported findings on the effect of prenatal education on the occurrence of vaginal birth. The combined results of these studies showed that women who participated in prenatal education were approximately twice as likely to have a vaginal birth as those who did not participate (OR: 2.00, 95%CI: 1.51–2.65, z = 4.82, p < 0.001, I^2^ = 0, p < 0.001; Fig. 5a). Similarly, the combined results of ten studies showed that the preference for vaginal birth was approximately three times higher in women who received prenatal education compared to those who did not (OR: 2.87, 95%CI: 1.69–4.89, z = 3.89, p = 0.001, I^2^ = 56, Fig. 5b).

### Publication bias between the studies

**Fig. 6:**
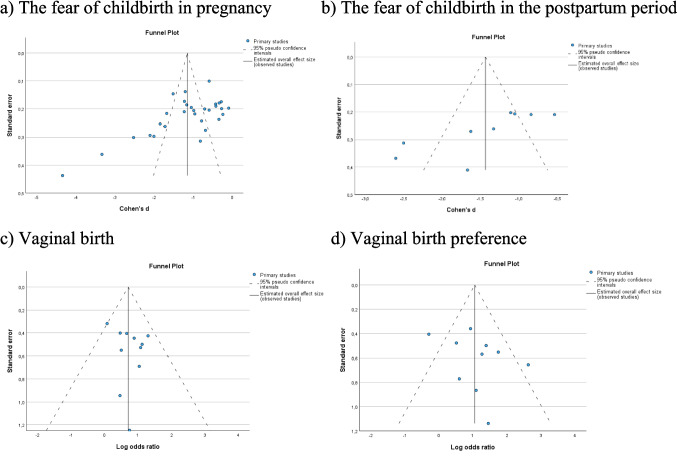
Publication bias for fear of childbirth in the pregnancy and postpartum period, vaginal birth, and vaginal birth preference

During the meta-analyses of this study, publication bias was examined with Funnel plot and Egger's regression-based test for the data sets created for fear of childbirth in pregnancy and postpartum period and vaginal delivery and vaginal delivery preference. In the analyses, it was determined that publication bias was statistically significant for fear of childbirth during pregnancy and postpartum period (t = −5.308, p < 0.001; t = −3.739, p = 0.007, respectively) and insignificant for vaginal delivery and vaginal birth preferences (t = 0.877, p = 0. 403; t = 1.062, p = 0.319, respectively) (Fig. 6).

### Certainty of evidence

The certainty of evidence was low for fear of childbirth during pregnancy, moderate for fear of childbirth in the postpartum period, and high for vaginal birth and preference for vaginal birth (Table 2).

**Table 2:** GRADE Summary of findings: Prenatal education compared to usual care for the fear of childbirth and birth type

**Intervention:** Prenatal education **Comparison:** usual care						
Outcomes	Anticipated absolute effects^*^ (95% CI)		Relative effect (95% CI)	№ of participants (studies)	Certainty of the evidence (GRADE)	Comments
	**Risk with usual care**	**Risk with prenatal education**				
The fear of childbirth in pregnancy	-	SMD **1.12 lower** (1.36 lower to 0.88 lower)	-	3523 (14 RCTs 16 quasi-experimental)	⨁⨁◯◯ Low^a,b,c^	Prenatal education may reduce the fear of childbirth in pregnancy
The fear of childbirth in the postpartum period	The mean the fear of childbirth in the postpartum period was **0**	MD **24.35 lower** (32.08 lower to 16.63 lower)	-	716 (7 RCTs 2 quasi-experimental)	⨁⨁⨁◯ Moderate^a,c^	Prenatal education may reduce the fear of childbirth in the postpartum period
Vaginal birth	534 per 1.000	**696 per 1.000** (633 to 752)	**OR 2.00** (1.51 to 2.65)	1009 (7 RCTs 4 quasi-experimental)	⨁⨁⨁⨁ High^a^	Prenatal education increases the probability of vaginal birth
Vaginal birth preference	736 per 1.000	**889 per 1.000** (825 to 932)	**OR 2.87** (1.69 to 4.89)	1201 (6 RCTs 4 quasi-experimental)	⨁⨁⨁⨁ High^a^	Prenatal education increases the likelihood of vaginal birth preference

^*^The risk in the intervention group (and its 95% confidence interval) is based on the assumed risk in the comparison group and the **relative effect** of the intervention (and its 95% CI)

**CI:** confidence interval; **MD:** mean difference; **OR:** odds ratio; **SMD:** standardised mean difference

GRADE Working Group grades of evidence

High certainty:we are very confident that the true effect lies close to that of the estimate of the effect

Moderate certainty:we are moderately confident in the effect estimate: the true effect is likely to be close to the estimate of the effect, but there is a possibility that it is substantially different

Low certainty:our confidence in the effect estimate is limited: the true effect may be substantially different from the estimate of the effect

Very low certainty:we have very little confidence in the effect estimate: the true effect is likely to be substantially different from the estimate of effect

**Explanations**

a. We downgraded by one level due to risk of bias arising from the quality of evidence (randomization process, confounding and deviations from intended intervention)

b. We downgraded by one level due to the structural characteristics of the instruments used to measure fear of childbirth

c. Publication bias was found to be statistically significant

## Discussion

In the present systematic review and meta-analysis based on the results of previous studies, the identified results of 28 studies conducted in different countries are presented to determine the effects of prenatal education on the fear of childbirth, vaginal birth rates and vaginal birth preferences. The results of the study are valuable in that they reveal comprehensive and up-to-date information regarding the effectiveness of prenatal education, which can enable the development of prenatal follow-up and health care services.

The findings of this study indicate that prenatal education is effective in reducing the fear of childbirth in both pregnancy and the postpartum period. Consistent with the results of this study, Alizadeh-Dibazari et al. (2023) in their systematic approach and meta-analysis of eleven studies report from their findings that prenatal education in addition to routine prenatal care could reduce the fear of childbirth among pregnant women. Similarly, another systematic review and meta-analysis on the effect of psych-education on the fear of childbirth and postpartum outcome reports a significant difference in the level of the fear of childbirth between pooled intervention group and pooled control group (Yıldız Karaahmet et al. 2023). In the present meta-analysis, various kinds of prenatal education such as online education, psychoeducation, companion-integrated education, cognitive-behavioural education, simulation-based education and even education specifically aimed at reducing the fear of childbirth are encountered (Akın et al. 2018; Dai et al. 2021; Firouzan et al. 2020; Khojasteh et al. 2022; Munkhondya et al. 2020; Uludağ et al. 2022). Nonetheless since the aim of this study was to determine the effect of prenatal education in general, it is unclear which kind of prenatal education, in particular, is most effective and hence most recommendable. More studies investigating the efficacy of the various kinds of prenatal education are therefore recommended.

There was significant heterogeneity among the prenatal education programs implemented in the studies included in this meta-analysis in terms of their type, content, and duration. A wide variety of additional approaches were integrated into prenatal education, including psychodrama, hypnobirthing, simulation-based education, psychoeducation (including solution-focused and cognitive-behavioural approaches), companion-supported programs, health literacy modules, Pilates-supported approaches, online education, and mindfulness-based birth and parenting programs. However, the meta-regression analysis revealed that prenatal education, either alone or in combination with these additional methods, did not have a consistent effect on pregnancy outcomes related to fear of childbirth. Similarly, previous meta-analyses reported that various prenatal education methods were generally effective in reducing fear of childbirth, although the certainty of evidence varied depending on the type of intervention (Alizadeh-Dibazari et al. 2023, 2024; Webb et al. 2021). Studies comparing the effectiveness of different educational methods have also reported that these interventions were similarly effective in reducing fear of childbirth (Khojasteh et al. 2022; Van der Meulen et al. 2023). Furthermore, Alizadeh-Dibazari et al. (2024) found that key variables such as maternal age, sample size, duration of prenatal education, and number of sessions did not significantly influence the outcomes related to fear of childbirth. These results are important because they demonstrate that prenatal education can effectively reduce fear of childbirth, but the magnitude of its effect may depend on variables such as the structural characteristics of the program, its scope and duration, and the characteristics of both educators and participants.

In this meta-analysis, it was determined that various measurement tools were used in assessing the fear of childbirth in pregnancy, but these measurement tools did not affect the effect size obtained regarding the fear of childbirth. The influence of the measurement tools on the results for the fear of childbirth has been reported elsewhere and is probably due to the lack of a standard for the diagnosis of the fear of childbirth as well as the way the tools are used (Richens et al. 2018). According to Richens et al. (2018), the Fear of Birth Scale is effective and more practical for measuring the fear of childbirth compared to W-DEQ which lengthy and hence less preferred in clinical settings. There is the need for a consensus on the definition of the fear of childbirth for more comparable results and standardised diagnosis.

In this systematic review, the certainty of evidence was low for fear of childbirth during pregnancy, moderate for fear of childbirth in the postpartum period and high for vaginal birth and preference for vaginal birth. Additionally, in the present study, the fear of childbirth during pregnancy did not vary based on the design of the studies included in the review, during the COVID-19 pandemic period, the country of the studies and data collection tools. There are mixed reports regarding the effect of the COVID-19 pandemic on the fear of childbirth; while some studies report that COVID-19 did not have any effect on the rates of fear of childbirth, some rates increased rates and some even suggest decreased rates (Aksoy et al. 2023; Dymecka et al. 2021; Thayer et al. 2023; Zilver et al. 2022). Lukasse et al. (2014) suggested from their study that the fear of childbirth was prevalent at similar rates in the six European countries included in their study. On the contrary, a systematic review analysing studies from nine countries concluded that although seven of the included countries used the same scale (W-DEQ), the rates of severe fear of childbirth in these countries varied significantly (Nilsson et al. 2018). The wide discrepancies between the current study and the studies mentioned could be attributed to the use of differing scales and research designs.

The results of the present meta-analysis showed that prenatal education increased the likelihood of vaginal birth and the preference for vaginal birth approximately twofold and threefold respectively. A strong positive correlation has been established between the fear of childbirth and increased caesarean rates, especially caesarean on maternal request (Elgzar et al. 2023; Yin et al. 2024). So, it is not surprising that prenatal education which has been found to reduce the fear of childbirth could reverse or prevent caesarean rates and therefore increase vaginal birth and the preference for it (Gao et al. 2022; World Health Organization 2021). Yıldız Karaahmet et al. (2023) revealed from their meta-analysis that preference for caesarean birth significantly reduced after pregnant women had received phych-education. Furthermore, a randomised controlled trial reported a significant increased preference both for physiologic birth and vaginal delivery (Masoumi et al. 2016).

### Strengths and limitations of the study

The strengths of the study include the fact that the studies examined in this meta-analysis have extensive scanning resources, the studies are up to date, they were conducted in different countries and most of the studies had a low risk of bias. Moreover, the large combined total sample size in the meta-analysis (3073) and the use of different analyses such as grade and meta-regression are more strengths of the research; these factors also strengthen the results of the study. However, the fact that the majority of studies were conducted in Turkey and Iran and included only a limited number of other countries restricts the generalizability of the findings. In addition, variations in the measurement tools used to assess fear of childbirth, differences in the structural characteristics of the educational programs implemented, the high level of heterogeneity among studies in the meta-analyses related to fear of childbirth, and the presence of significant publication bias may also represent limitations that weaken the strength of the evidence obtained in this study. Therefore, to avoid the effect of heterogeneity between the studies, the random effect model was chosen in the relevant meta-analyses.

## Conclusions

This meta-analysis revealed that birth preparation training is effective in reducing birth-related fears and increasing vaginal birth occurrences and vaginal birth preferences. These results are also supported by previously conducted research. Based on these results, it may be recommended that healthcare managers and practitioners reorganize prenatal follow-up and healthcare services to ensure that all pregnant women receive prenatal education. Moreover, standardized training programs containing minimum content appropriate for the health service delivery and socio-cultural characteristics of the countries could be prepared and implemented. Additionally, meta-analysis studies including current research that will support the results of this study, experimental studies that test the effectiveness of more applicable prenatal education programs standardized according to the current conditions of the countries, and qualitative studies examining women's experiences with prenatal education programmes may be recommended.

## Data Availability

Additional data on the study can be provided by the corresponding author if requested.

## Abbreviations

CI: Confidence interval
COVID-19: Coronavirus Disease 2019
CAS: Childbirth Attitude Scale
FBPQ: Fear and Birth Preference Questionnaire
FOC: Fear of childbirth
GRADE: The grading system of recommendations, assessment development and evaluation
MD: Mean difference
SMD: Standardized mean difference
RCT: Randomized controlled trial
PRISMA: Preferred Reporting Items for Systematic Reviews and Meta-Analyses
W-DEQ: Wijma delivery expectancy/experience questionnaire
